# Longitudinal Risk Prediction of Chronic Kidney Disease in Diabetic Patients Using a Temporal-Enhanced Gradient Boosting Machine: Retrospective Cohort Study

**DOI:** 10.2196/15510

**Published:** 2020-01-31

**Authors:** Xing Song, Lemuel R Waitman, Alan SL Yu, David C Robbins, Yong Hu, Mei Liu

**Affiliations:** 1 University of Kansas Medical Center Department of Internal Medicine, Division of Medical Informatics Kansas City, KS United States; 2 University of Kansas Medical Center Division of Nephrology and Hypertension and the Kidney Institute Kansas City, KS United States; 3 University of Kansas Medical Center Diabetes Institute Kansas City, KS United States; 4 Jinan University Big Data Decision Institute Guangzhou China

**Keywords:** diabetic kidney disease, diabetic nephropathy, chronic kidney disease, machine learning

## Abstract

**Background:**

Artificial intelligence–enabled electronic health record (EHR) analysis can revolutionize medical practice from the diagnosis and prediction of complex diseases to making recommendations in patient care, especially for chronic conditions such as chronic kidney disease (CKD), which is one of the most frequent complications in patients with diabetes and is associated with substantial morbidity and mortality.

**Objective:**

The longitudinal prediction of health outcomes requires effective representation of temporal data in the EHR. In this study, we proposed a novel temporal-enhanced gradient boosting machine (GBM) model that dynamically updates and ensembles learners based on new events in patient timelines to improve the prediction accuracy of CKD among patients with diabetes.

**Methods:**

Using a broad spectrum of deidentified EHR data on a retrospective cohort of 14,039 adult patients with type 2 diabetes and GBM as the base learner, we validated our proposed Landmark-Boosting model against three state-of-the-art temporal models for rolling predictions of 1-year CKD risk.

**Results:**

The proposed model uniformly outperformed other models, achieving an area under receiver operating curve of 0.83 (95% CI 0.76-0.85), 0.78 (95% CI 0.75-0.82), and 0.82 (95% CI 0.78-0.86) in predicting CKD risk with automatic accumulation of new data in later years (years 2, 3, and 4 since diabetes mellitus onset, respectively). The Landmark-Boosting model also maintained the best calibration across moderate- and high-risk groups and over time. The experimental results demonstrated that the proposed temporal model can not only accurately predict 1-year CKD risk but also improve performance over time with additionally accumulated data, which is essential for clinical use to improve renal management of patients with diabetes.

**Conclusions:**

Incorporation of temporal information in EHR data can significantly improve predictive model performance and will particularly benefit patients who follow-up with their physicians as recommended.

## Introduction

### Background

With the rapid development in digitization of health care data, the modern electronic health records (EHRs) hold considerable promise for driving scientific advances in various aspects of biomedicine through the utilization of machine learning techniques. EHRs contain not only diverse clinical data elements that can better describe a patient’s overall health status but also rich longitudinal data of patients that serve as a critical source for understanding the evolution of disease and management of chronic conditions. Developing accurate risk prediction models to drive timely initiation of appropriate therapies and monitoring is of paramount importance for conditions that have a substantial public health impact and can benefit greatly from early intervention.

Chronic kidney disease (CKD), especially CKD attributed to diabetes, that is, diabetic kidney disease (DKD), certainly falls within this category [1]. DKD is one of the most frequent and dangerous microvascular complications in diabetes mellitus (DM) that affects about 20% to 40% of patients with type 1 or type 2 DM [2]. It is the leading cause of end-stage renal disease (ESRD), which accounts for approximately 50% of the cases in the developed world with major public health and economic implications [3]. Therefore, annual screening is recommended for patients with type 1 and type 2 diabetes [4,5], which in turn has two implications: (1) there is a better chance for us to observe more regular and meaningful temporal patterns among these patients, and (2) an effective model for predicting the risk of DKD in the following year can be more beneficial for patients who are compliant to the annual check protocol because this allows implementation of early preventive measures.

### Related Work

The effective use of temporal EHR data for predictive modeling remains a challenge owing to its highly variable sampling rates across different groups of patients (eg, patients may not follow the annual check protocol and only visit the hospital for critical health events) and distinct data types (eg, vital signs are noted hourly during inpatient encounters, whereas laboratory tests and medications are recorded when clinicians order them, and demographic data are more stable). Attempts have been made to handle temporal information in a variety of clinical applications. One approach involves representing the time series of clinical features with a single heuristic value (eg, taking the latest value or the trend [6] or shrinking to a weighted sum of values with the *weights* determined by the timestamps [7,8]). Another approach is to preserve the underlying sequential order by mapping the time series into temporal patterns (eg, knowledge-based temporal abstraction or hidden Markov chains [9,10]) or symbolic representations (eg, the Symbolic Aggregate approXimation based on Gaussian quantiles and the temporal discretization for classification [11,12]). Moreover, deep learning techniques such as recurrent neural networks, in particular, long- and short-term memory and Gated recurrent units, have contributed to model temporal events [13-15]. However, it has also been reported in the corresponding work that many such approaches could suffer from high data sparsity or *informative missingness* and insufficient training data.

In the prediction of kidney-related events, single-value abstraction is the most popular approach for its simplicity but at the expense of reduced temporal granularity. For example, in the ADVANCE prospective study for diabetic nephropathy, only baseline values of selected labs and vitals are used in a Cox proportional survival model [16]. A multivariate Cox proportional survival model was developed for predicting ESRD based on mean- and variation-abstractions of repeated glycated hemoglobin (HbA_1c_), creatinine, and blood pressure measurements [17]. More sophisticated use of temporal EHRs has also been studied, many of which were targeted at severe or acute kidney-related events. A Bayesian multiresolution hazard model for predicting CKD progression from stage III to stage IV attempted to capture temporal patterns by associating variables with piece-wise hazard increments at different time windows [18], whereas an independent Markov process modeled the underlying sequential latent states for predicting the transition from CKD stage III to stage IV [19]. A multitask linear model enabled knowledge transfer from one time window to another in the prediction of short-term renal function loss [20], and a tree-based discrete-survival-like gradient boosting machine (GBM) predicting acute kidney injury in inpatients allowed the features and their association with outcome to be time variant and showed excellent performance [21]. However, all of the aforementioned approaches require moderate to high manual effort on feature preselection and curation, which not only limits the scalability of the predictive models but also discards considerable amount of information in each patient’s records [15]. In addition, the complexity of EHR data often violates the linearity and independence assumptions for survival and linear models, resulting in worse predictions and impaired generalizability.

### Objectives

In this study, we propose a new approach for incorporating the temporal information in medical history of patients with diabetes to further improve the predictive model for evaluating their risk of renal complication in the next year. Because of its robustness, efficiency, and established efficacy in the prediction of kidney events [21], we chose GBM as the base learner and augmented it with schemes to continuously update its learning results based on new patient inputs over a full breadth of EHR data on a yearly basis, named *Landmark-Boosting*. Here, the *landmark* time refers to an unbiased reference point (eg, *t* years since the onset of DM) at which we want to construct stagewise prediction models and make dynamic risk predictions using information collected up to that time [22,23]. The final prediction model is then an ensemble of individual boosting models trained at each landmark time *a* *priori*.

## Methods

### Definition of Diabetes

We adopted the Surveillance, Prevention, and Management of Diabetes Mellitus definition of diabetes in this study. Diabetes was defined based on the following: (1) the use of glucose-lowering medications (insulin or oral hypoglycemic medications); or (2) level of HbA_1c_ of 6.5% or greater, random glucose of 200 mg/dL or greater, or fasting glucose of 126 mg/dL on at least two different dates within 2 years; or (3) any two type 1 and type 2 DM diagnoses been given on 2 different days within 2 years; or (4) any two distinct types of events among (1), (2), or (3); and (5) excluding any gestational diabetes (temporary glucose rise during pregnancy) [24]. DM onset time was defined as the first occurrence of any events from (1) through (5).

### Definition of Diabetic Kidney Disease

DKD was defined as diabetes with the presence of microalbuminuria or proteinuria, impaired glomerular filtration rate (GFR), or both [25,26]. Microalbuminuria was defined as albumin-to-creatinine ratio (ACR) being 30 mg/g or greater, and similarly, proteinuria was defined as urine protein-to-creatinine ratio being 30 mg/g or greater [25,26]. Impaired GFR was defined as the estimated GFR (eGFR), an age-, gender-, race-adjusted serum creatinine concentration based on the modification of diet in renal disease equation [27] being less than 60 mL/min/1.73 m^2^.

### Study Cohort

**Figure 1:**
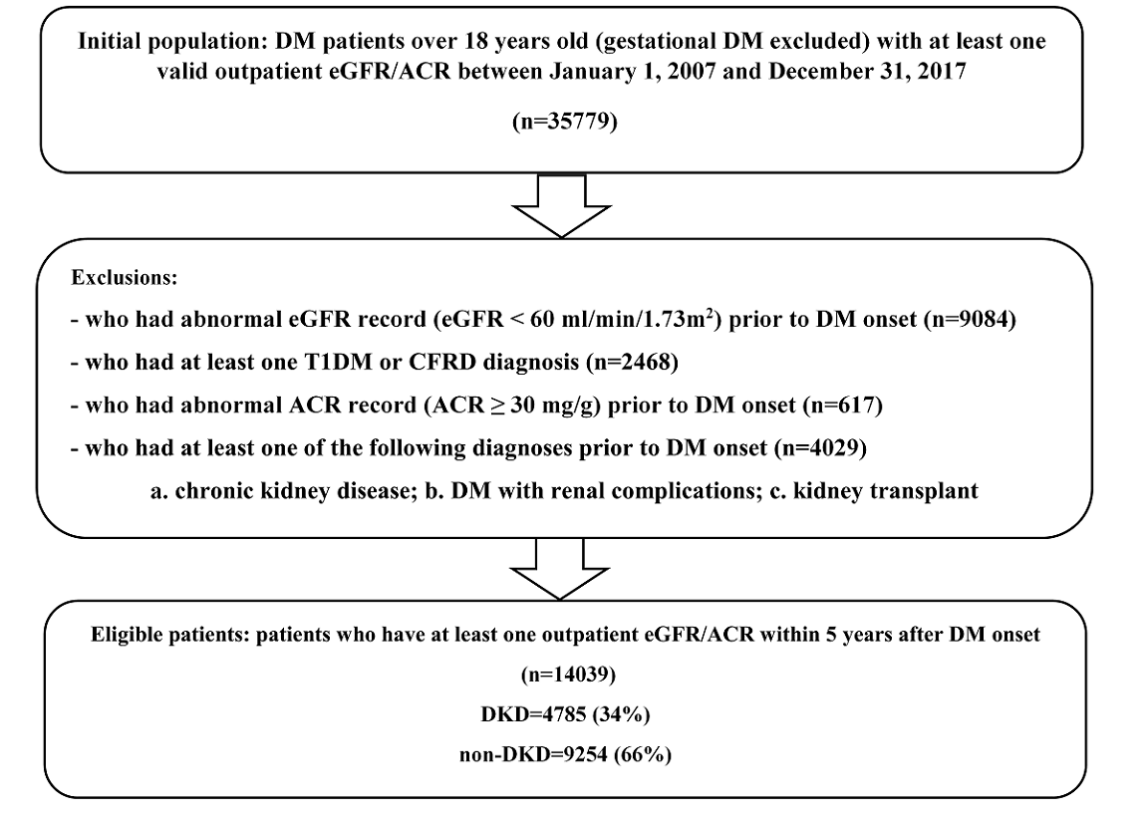
Study cohort inclusion and exclusion. Note that the counts of exclusions do not necessarily add up to the difference between the initial and final population, as 1 patient could satisfy multiple exclusion criteria. ACR: albumin-to-creatinine ratio; DKD: diabetic kidney disease; DM: diabetes mellitus; EGFR: estimated glomerular filtration rate.

The study constructed a retrospective cohort using deidentified EHR and billing data from November 2007 to December 2017 in the University of Kansas Medical Center’s integrated clinical data repository Healthcare Enterprise Repository for Ontological Narration (HERON) [28]. The study did not require approval from the institutional review board because data used met the deidentification criteria specified in the Health Insurance Portability and Accountability Act Privacy Rule. The HERON Data Request Oversight Committee approved the data request. As shown in Figure 1, a total of 35,779 adult patients with nongestational DM (age≥18 years) who had at least one valid eGFR or ACR record at an outpatient encounter were eligible for this study so that they could be identifiable as DKD present or not. We excluded patients presenting with any type 1 DM or cystic fibrosis–related diabetes diagnoses over their observation period and those who had kidney disease manifestation (eg, CKD diagnosis, low eGFR, or microalbuminuria) before the onset of DM. The case group included all DKD patients with their DKD onset time, or end point, defined as the first time of their abnormal eGFR or ACR. The control group was defined as patients with DM whose eGFR values were always above or equal to 60 mL/min/1.73 m^2^ and had never had microalbuminuria, with their end point defined as the last time of their normal eGFR or ACR. Finally, 14,039 patients were included in the final cohort with 4785 (34.08%) patients with DKD.

### Clinical Variable Extraction

According to our data, the heuristic time between 2 adjacent outpatient eGFR or ACR labs is on average 1 year per patient. Thus, for a patient *i,* a sequence of time-stamped examples (ie, DKD statuses, 1 for DKD and 0 for non-DKD), is identified based on their last outpatient eGFR or ACR collected annually, denoted as {y_i_^t^}_t_^T^. Note that a patient may be missing eGFR/ACR during certain years, and we kept the corresponding DKD status as *NA* without any imputation. For example, the outcome sequence for a patient can be (0, NA, 1), which can be interpreted, respectively, as “the patient did not have DKD the same year as DM onset, but cannot determine DKD status for the second year, and had DKD onset in the third year.”

Each patient was then represented by collecting 15 common types of clinical observations from HERON [28] (Table 1). Each category is a mixture of categorical and numerical data elements. Numeric values were used for laboratory tests and vital signs, whereas binary indicator variables were used for categorical features. In addition, we abstracted the Medication variables at the Semantic Clinical Drug Form or Semantic Clinical Brand Form level and Diagnoses variables at the International Classification of Diseases (ICD)-9 or 10 code level [29]. We further decomposed clinical features into more meaningful pieces according to (1) different sources of a diagnosis (ie, billing diagnoses or EHR problem list diagnoses), (2) different aspects of a medication fact (ie, drug refill or drug amount), (3) different types of encounters where a procedure was ordered or performed (ie, inpatient or outpatient), and (4) different states of an alert (ie, fired or overridden). These data elements were extracted from our institutional EHR and had been explicitly incorporated in our data warehouse as an additional i2b2-specific attribute called *modifier* [30]. Among the initial 22,331 distinct features available for our study cohort, 15,707 (70%) were only recorded for <1% of the patients, which we excluded to reduce data sparsity.

**Table 1:** Integrated data repository data domain categories.

Domain	Descriptions	Data type	Number of eligible features^a^	Patients^b^, n (%)
Alerts	Includes drug interaction, dose warnings, drug interactions, medication administration warnings, and best practice alerts	Binary	531	11,848 (84.39)
Allergy	Includes documented allergies and reactions	Binary	49	5044 (35.93)
Demographics	Basic demographics such as age, gender, race, etc, as well as their reachability, and some geographical information	Binary/numeric	10	14,039 (100.00)
Diagnoses	Organized using ICD^c^-9 and ICD-10 hierarchies. Intelligent Medical Objects interface terms are grouped to ICD-9 and ICD-10 levels. Diagnosis resources are further separated by source of the assignment (eg, EMR^d^, professional billing, technical billing, and registry).	Binary	1186	12,616 (89.86)
History	Contains family, social (ie, smoking), and surgical history from the EMR, as well as engineered features such as number of distinct clinical facts and clinical fact increments since last collection point	Binary/numeric	155	12,178 (86.74)
Laboratory tests	Results of a variety of laboratory tests, including cardiology and microbiology findings. Note that the actual laboratory values are used in modeling, if available.	Binary/numeric	685	11,990 (85.40)
Medications	Includes dispensing, administration, prescriptions, as well as home medication reconciliation at the University of Kansas Hospital grouped at Semantic Clinical Drug Form or Semantic Clinical Brand Form level. Medication resources are further separated by types of medication activity.	Binary	1205	8295 (59.09)
Procedures	Includes Current Procedural Terminology professional services and inpatient ICD-9 billing procedure codes.	Binary	560	12,460 (88.75)
Orders	Includes physician orders for nonmedications, such as culture and imaging orders from the EMR.	Binary	1053	12,460 (88.75)
Vizient (billing)	(formerly University Health System Consortium) Includes both billing classifications such as Diagnostic Related Groups, comorbidities, discharge placement, length of stay, and national quality metrics.	Binary	657	3619 (25.78)
Visit details	Includes visit types, vital signs collected at the visit, discharge disposition, and clinical services providing care from both EMR and billing.	Binary/numeric	474	13,671 (97.38)

^a^This does not include all distinct concepts from the entire Healthcare Enterprise Repository for Ontological Narration system; it only includes the total number of distinct features that had ever been recorded for at least one patient in the study cohort.

^b^This is the number of patients who have at least one observation during any time window recorded from the corresponding data domain.

^c^ICD: International Classification of Diseases.

^d^EMR: electronic medical record.

**Figure 2:**
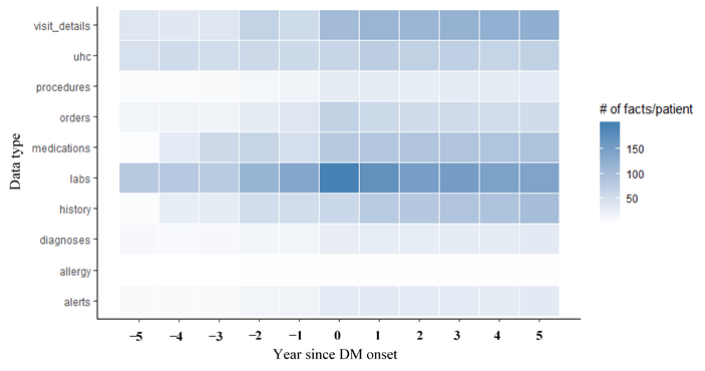
Clinical feature densities across data types. Each row corresponds to the average number of distinct clinical facts per patient for a certain type of clinical data over 5 years before and after DM onset. The darker the region is, the more distinct facts have been recorded for patients on average within the corresponding time window. DM: diabetes mellitus; UHC: University HealthSystem Consortium.

In Figure 2, we illustrated the feature densities over time across different data types. Each row corresponds to the average number of distinct clinical facts per patient for a data type over 5 years before and after DM onset. An evident heterogeneity of clinical activities before and after DM onset can be observed. For example, lab frequencies are much higher in the first 2 years of DM onset, with visits becoming more frequent after DM onset.

In Table 2, we characterized the temporal variations by estimating the between-observation time, or observation intensity, for each data type and observed that the between-patient irregularity of sampling rates is significantly different from within-patient (*P*<.001) based on the analysis of variance tests, except for demographics, suggesting varying degrees of health care exposure across patients and over time.

**Table 2:** Clinical observation intensity.

Data type^a^	Mean time lapses (days)	Within-patient standard deviation (days)	Between-patient standard deviation (days)	*P* value
Alerts	67	93	146	<.001
Allergy	169	158	214	<.001
Diagnoses	87	105	133	<.001
History	184	230	872	<.001
Laboratory tests	107	122	175	<.001
Medications	70	70	137	<.001
Procedures	74	99	132	<.001
Orders	81	95	127	<.001
Vizient	228	189	304	<.001
Visit details	36	61	70	<.001

^a^Demographics are not included as they are unique at the patient level.

### Experimental Design

For the clinical task of predicting DKD risk over the next year, we first randomly divided the 14,039 patients into training set (80%) for model development and validation set (20%) for performance evaluations. To simulate a more realistic clinical scenario and account for the bias caused by varying degrees of health care exposure over time, we stepped forward through patients’ time course and built prediction models at each landmark time, that is, every full year since DM onset, for rolling predictions of 1-year DKD risk. As such, individuals may contribute to or be tested by one or more prediction models, depending on their eligibility at the landmark time.

### Gradient Boosting Machine

We chose GBMs as the baseline training model, which were then combined with four different approaches to incorporate temporal data. GBM is a family of powerful machine-learning techniques that have shown considerable success in a wide range of practical applications [31-36]. We chose GBM as the base learner for its robustness against high dimensionality and collinearity and also because it embeds feature selection scheme within the process of model development [37]. To better control overfitting, we tuned the hyperparameters (depth of trees: 2-10; learning rate: 0.01-0.1; minimal child weight: 1-10; number of trees is determined by early stopping, ie, if the holdout area under the receiver operating curve [AUROC] had not been improved for 100 rounds, then we stopped adding trees) within the training set using 10-fold cross-validations.

### Missing Values

Missing values were handled in the following fashion: for categorical data, a value of 0 was set for missing, whereas for numerical data, a *missing value split* was always accounted for, and the *best* imputation value can be adaptively learned based on the improvement in training AUROC at each tree node within the ensemble [38]. For example, if a variable *X* takes values (0, 1, 2, 3, NA, and NA), where *NA* stands for missing, the following two decisions will be made automatically at each split for each tree: (1) should we split based on *missing or not*? and (2) if we split based on values, for example, >1 or ≤0, should we merge the missing cases with the bin of >1 or ≤0?

### Evaluation Metrics

We used AUROC and area under precision recall curve (AUPRC) to compare the overall prediction performance, with the latter known to be more robust to imbalanced datasets. In addition, we characterized calibration by the observed-to-expected outcome ratio (O:E), which measures agreement between the predicted and observed risk on average across observations. By treating testing examples with predicted probability of outcome in the top 40th percentile as positive cases, we made fair performance comparisons among different methods and further examined the model’s ability in detecting positive vs negative cases by reporting the sensitivity, specificity, positive predictive values (PPVs), and negative predictive values.

### Temporal Information Incorporation

**Figure 3:**
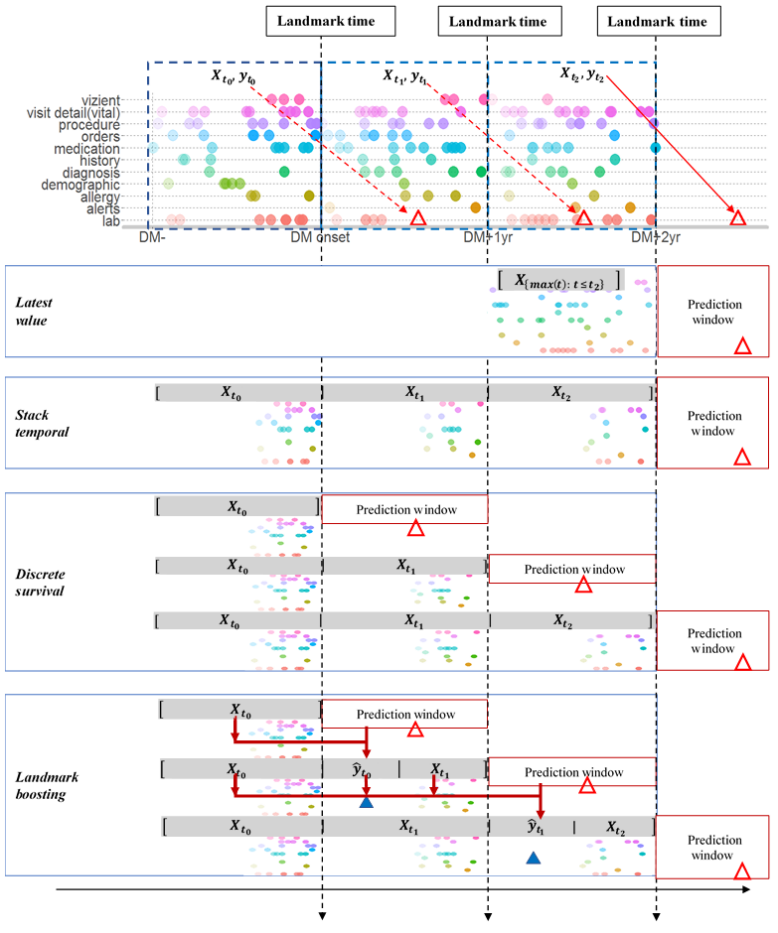
Illustration of the temporal approaches, which are Latest-Value, Stack-Temporal, Discrete-Survival, and Landmark-Boosting from top to bottom. Different colors of circles represent different types of clinical data. Red triangles represent real values of the outcome (ie, diabetic kidney disease (DKD) or non-diabetic kidney disease in the following prediction window). Blue triangles represent predicted outcome based on clinical features presented in the previous observation window. Xti denotes all available clinical features collected strictly before landmark time ti (ie, number of full years since DM onset). yti denotes real label of DKD onset after within the prediction window (ti, ti+1). DM: diabetes mellitus.

Figure 3 depicts the four different approaches explored in this study for handling temporal EHR data: *Latest-Value* provides the most straightforward way to aggregate repeatedly measured variables; *Stack-Temporal* attempts to differentiate the effects of the same variable associated with different timestamps; and *Discrete-Survival* allows survival analysis model to be created by using binary classifier, which effectively enhances the chronical relationship between the predictors and the outcome. Landmark-Boosting is our proposed model motivated by the boosting method, which is designed to ensemble identification trees by learning over time. Each of the approaches is discussed in detail in the following sections.

#### Latest-Value Approach

In this approach, we simply collect the last observed value before each landmark time for each predictor across all time windows (Figure 3) [16]. The Latest-Value approach is time agnostic, which implies it only retains the information about existence of certain predictors at the patient level. For example, the latest creatinine recorded for patient A can be 1 month ago but 1 year ago for patient B, which will be treated equally by this approach.

#### Stack-Temporal Approach

Given the variables for all time windows T, the Stack-Temporal approach concatenates the variable from all windows to represent patient *x*_i_ using p-dimensional vector, where p=number of variables x T (Figure 3) [20]. One of the disadvantages of this approach is that the feature dimensionality increases proportionally to T, which may lead to worse prediction performance because of overfitting.

#### Discrete-Survival Approach

The Discrete-Survival approach simulates a discrete-time survival framework by separating the full course of patient’s medical history into *L* nonoverlapping yearly windows, *L*=1,2,...*T*, with variables from *t*-1 to predict DKD risk in *t* (Figure 3) [21]. This approach assumes that examples from different time windows are independent of each other even if they may come from the same patient, which does not explicitly allow knowledge to be transferred from the previous time window to the next.

#### Landmark-Boosting Approach

**Figure 4:**
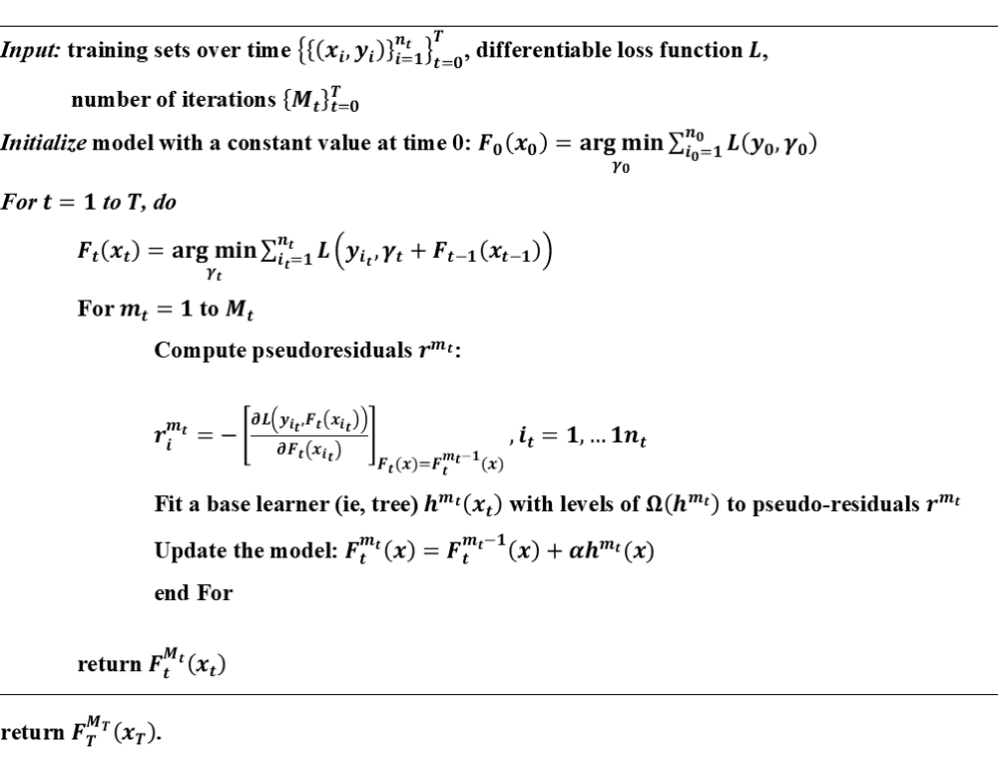
Pseudocode for landmark boosting algorithm. In this experiment, Mt (the number of trees at each iteration is set to 1000), α (learning rate), and Ω(hMt) (levels of each tree) are hyperparameters tuned by 10-fold cross-validation on the training dataset at each iteration.

To build the continuous learning mechanism, we developed a new method by extending the classical GBM to ensemble learners over time, that is, from one landmark time to the next (Figure 3). Specifically, we collected data *D_t_*={(*x_it_ , y_i_*)} with *i=1,2,…,N_t_* at each time window *t* and tried to solve the following optimization problem sequentially for all 1≤*t≤T,*

min *E_t|t-1_*[*L*(*y, F_t_* (*x_t_, F_t-1_*(*x_t-1_,y_t-1_*)))] (1)

where *F* represents the prediction function (ie, ensemble of trees), *L* represents the loss function (ie, logloss), and *E*_t/_*_t_*_-1_ stands for conditional expectation at time*t* using observed values at time *t*-1. In other words, we used the predicted probability from time *t*-1 as the baseline risk and ensembled new learners based on predictors updated at time *t*. Figure 4 presents the algorithm describing the detailed implementation steps.

## Results

### Cohort Characteristics

At each landmark time, the eligibility of a patient was determined by checking if a valid eGFR or ACR reading presented in the current time window and was neither DKD nor censored in the previous time windows. As shown in Table 3, the number of eligible patients dropped over time with an increasing DKD rate as a mixing result of cases dropping out or censored from last time.

There is a mild decreasing trend of age and race (white) proportion over the landmark times. In addition, we compared such case-mix shifts between training and testing sets and found no significant differences (Table 4).

**Table 3:** Case-mix shift over landmark time.

Landmark time (number of years since DM^a^ onset)	Eligible, n (%)	DKD^b^, n (%)	Age (years), mean (SD)	Sex (male), n (%)	Race (white), n (%)
0	10,705 (76.25)	1673 (15.63)	58 (13)	5229 (48.84)	7221 (67.45)
1	7755 (72.44)	1467 (18.92)	58 (13)	3782 (48.77)	5185 (66.86)
2	5689 (73.36)	1163 (20.44)	57 (13)	2734 (48.06)	3715 (65.30)
3	4113 (72.30)	914 (22.22)	56 (12)	2002 (48.67)	2671 (64.94)
4	3006 (73.09)	740 (25.73)	56 (12)	1480 (49.23)	1941 (64.57)

^a^DM: diabetes mellitus.

^b^DKD: diabetic kidney disease.

**Table 4:** Case-mix shift in training and testing sets.

Landmark time (number of years since DM^a^ onset)		Training (n=11,184)	Testing (n=2855)	*P* value^b^
**Eligible**				
	0	8524	2181	—^c^
	1	6174	1581	—
	2	4537	1152	—
	3	3254	859	—
	4	2366	640	—
**Diabetic kidney disease, n (%)**				
	0	1352 (15.86)	321 (14.72)	.19
	1	1174 (19.02)	293 (18.53)	.66
	2	952 (20.98)	211 (18.32)	.05
	3	732 (22.50)	182 (21.19)	.41
	4	586 (24.77)	154 (24.06)	.71
**Age (years), mean (SD)**				
	0	57.8 (13.1)	57.4 (13.1)	.98
	1	57.6 (12.8)	57.3 (12.7)	.98
	2	57.0 (12.6)	56.9 (13.1)	>.99
	3	56.4 (12.6)	57.1 (12.0)	.96
	4	56.1 (12.3)	56.7 (11.7)	.99
**Sex (male), n (%)**				
	0	4183 (49.07)	1046 (47.96)	.98
	1	3023 (48.96)	759 (48.01)	.98
	2	2208 (48.67)	526 (45.66)	.95
	3	1593 (48.96)	409 (47.61)	.98
	4	1173 (49.58)	307 (47.97)	.97
**Race (white), n (%)**				
	0	5776 (67.76)	1445 (66.25)	.97
	1	4145 (67.14)	1040 (65.78)	.97
	2	2975 (65.57)	740 (64.24)	.97
	3	2123 (65.24)	548 (63.79)	.95
	4	1541 (65.13)	400 (62.50)	.89

^a^DM: diabetes mellitus.

^b^*P* value is based on two-sample *t* test for age and two-sample proportion test for the other comparisons.

^c^The two-sample test is not applicable for the corresponding comparison.

### Prediction Performance

**Figure 5:**
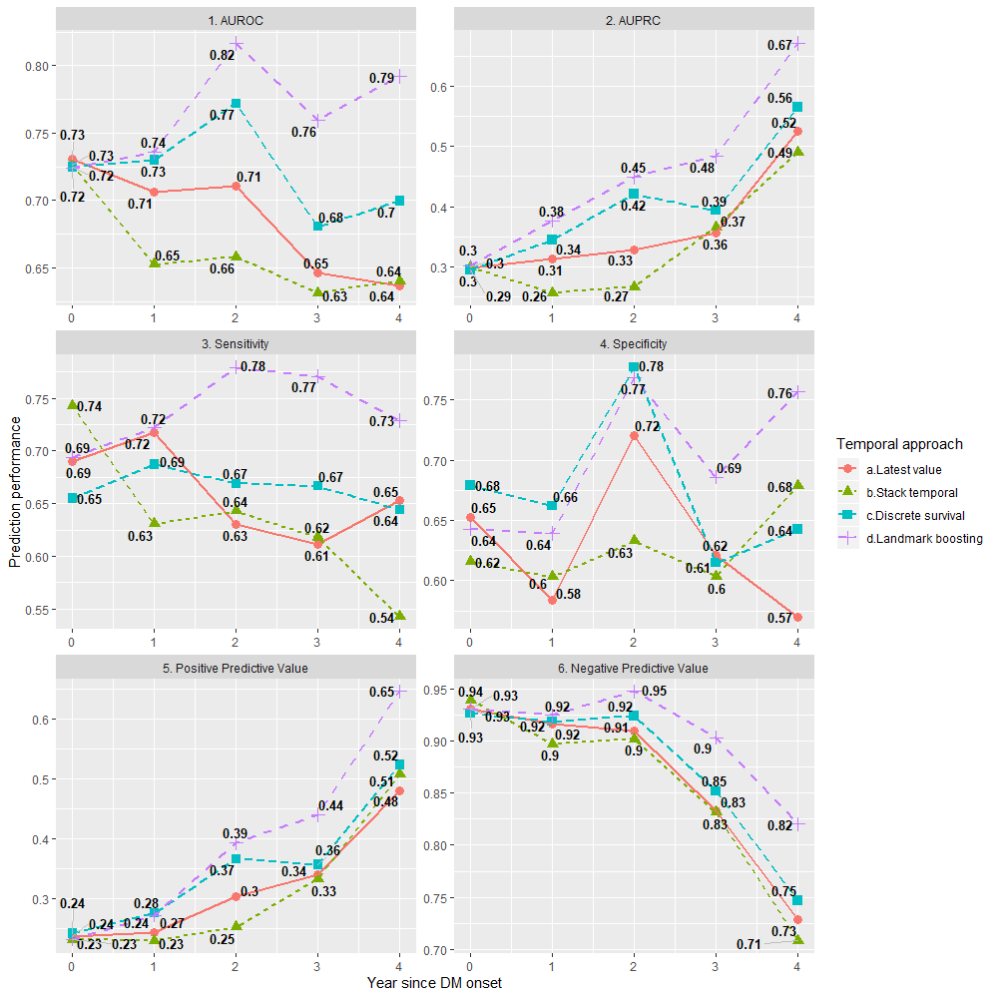
Performance comparisons among temporal approaches over landmark time. Area under receiver operating curve (AUROC) and area under the precision-recall curve (PRAUC) are first reported. For fair comparisons, sensitivity, specificity, positive predicted value, and negative predicted value are calculated by treating testing examples with predicted probability of outcome in the top 40th percentile as positive cases. Here, 95% bootstrap confidence intervals are reported for each metric at each landmark time (ie, full year since diabetes mellitus [DM] onset). The bootstrap confidence intervals are generated based on 30 bootstrapped samples, and used 2.5th percentile, 50th percentile, and 97.5th percentile to construct the confidence intervals for each metric.

Overall, the prediction results in Figure 5 showed that the proposed Landmark-Boosting model outperformed other temporal data representation methods with respect to all evaluation metrics. The Stack-Temporal approach always showed the worst performance, whereas the Latest-Value and Discrete-Survival approaches demonstrated competitive results. Only the Landmark-Boosting model had an increasing trend in AUROC over the years after DM onset, which peaked at =2 with value of 0.83 (95% CI 0.76-0.85). AUPRC showed a steadily increasing performance of all approaches over time, whereas the Landmark-Boosting model dominated at each landmark time and reached 0.75 (95% CI 0.65-0.80) at =4. Sensitivity declined slightly over time and achieved an optimal point at *t*=2 with the Landmark-Boosting model persistently outperforming others with a sensitivity of 83% (95% CI 79%-88%). In terms of specificity, Landmark-Boosting also outperformed others at each landmark time and achieved 78% (95% CI 74%-83%) at landmark time 4. Moreover, PPV improved over landmark time with the Landmark-Boosting approach showing the best performance reaching 67% (95% CI 57%-75%) at landmark time 4 (whereas the second-best model, Discrete-Survival, achieved 51% [95% CI 44%-57%]), translating to correct identification of 503 patients with DKD (whereas the second-best model only identified 383 patients with DKD).

**Figure 6:**
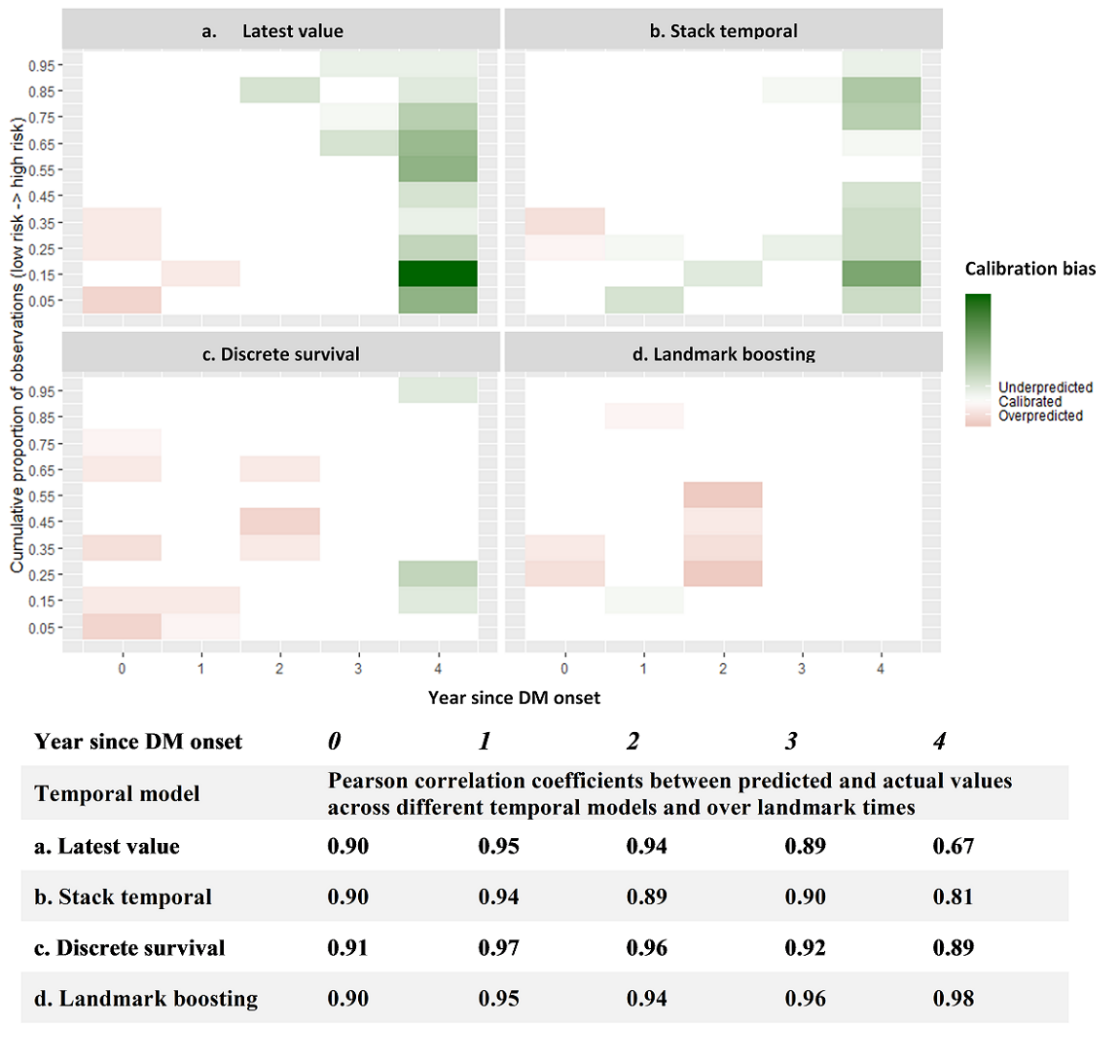
Calibration comparisons among temporal approaches over landmark time. Regions of calibration across the range of predicted probabilities, scaled by proportion of observations in each region and shaded by the magnitude of the within-region observed-to-expected ratio (O:E), with green suggests underprediction (ie, O:E significantly less than 1), and red suggests overprediction (ie, O:E significantly larger than 1). Pearson correlation coefficients between predicted and actual values over landmark times for each temporal model are included in the table below (the closer the coefficient is to 1, the better the predicted and actual values are linearly related). DM: diabetes mellitus.

Figure 6 presents regional calibration on the original predicted probability scale grouped into 20 bins. The *overpredicted* or *underpredicted* was defined as “the O:E ratio within a prediction bin that is significantly below or above 1 (*P* value<.05),” whereas the remaining cases were considered *calibrated*. Clearly, the Landmark-Boosting approach also dominated all other temporal methods on calibration, with a dip of overestimation for the group with moderate risk at *t*=2. Both Latest-Value and Stack-Temporal models underestimated the risk, especially at >2. Discrete-Survival model appeared to overestimate the risk at early years for the low-risk group but tended to underestimate the risk in later years.

### Case Study

**Figure 7:**
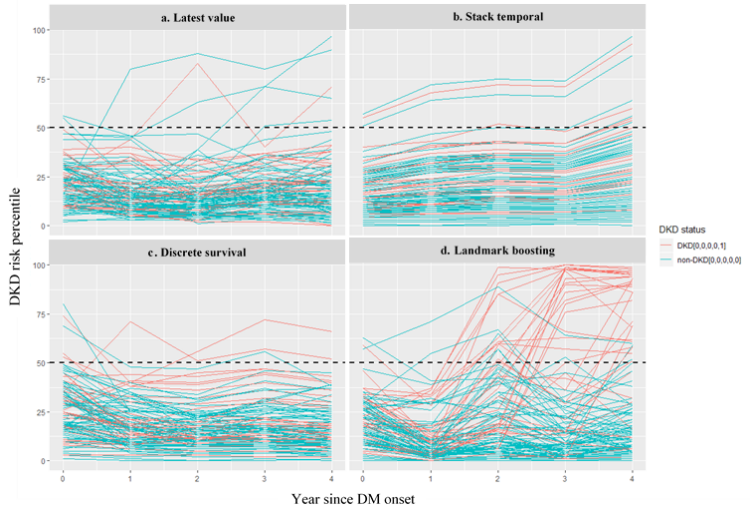
A visualization of predicted diabetic kidney disease (DKD) risk over landmark time. Risk percentiles (ie, normalized risk scores) against landmark time for a sample of patients. Each red line represents patient who finally progressed to DKD, whereas each green line represents patient who did not. DM: diabetes mellitus.

To closely examine the prediction change over time, we extracted a subset of 111 testing cases eligible at all five landmark times (ie, who had outcome sequence either like [0,0,0,0,0] or [0,0,0,0,1]) and plotted their predicted probability percentiles over years (Figure 7). We observed significant differences in the risk trajectory between patients with and without DKD depicted by the Landmark-Boosting method, with a much sharper increase of relative risk for most patients with DKD after year 1 and more obvious separation of risks over time. On the other hand, all other three methods suggested stable or even decreasing relative risk for patients with DKD over time, without much deviation from patients without DKD, with only a few exceptions.

## Discussion

### Principal Findings

The study results suggested that exploiting historical temporal EHR data in predictive models would significantly improve prediction performance, especially with our proposed Landmark-Boosting model. As demonstrated in Figure 5, the 4 different temporal models started with similar predictive power during the same year of DM onset but started to deviate along the landmark times. We observed a declining AUROC over time, with our proposed model being the only exception. One potential explanation is that the sensitivity of other three models may be affected by the upward case-mix shift (Table 3), that is, the models’ ability to detect positive cases was impaired. For example, the optimal sensitivity of Stack-Temporal model seemed to top at the beginning but suffered a severe drop over time without any significant improvement of specificity, which may be a result of potential overfitting caused by increasing dimensionality. Within the first 2 years, the Latest-Value model seemed to yield a competitive sensitivity against the Landmark-Boosting model while the latter exceled afterward, indicating the effect of continuous self-correction mechanism that began to manifest after the second year since DM onset. A local peak of specificity presenting at year 2 for all four models implied a change in their *interests* toward the non-DKDs; however, only the Landmark-Boosting model kept the balance by preserving a good sensitivity. In contrast with AUROC, which has been criticized as being susceptible to class imbalance [39], AUPRC demonstrated a steady trend of increase over landmark times for all temporal models, which was mainly attributable to PPV improvement, indicating that the signals from DKD samples may have become stronger over time, likely as a result of increasing DKD prevalence over the landmark years. Nonetheless, the proposed Landmark-Boosting model dominated the others and even showed increasing margins along landmark times. For instance, the Landmark-Boosting model identified 46, 36, and 120 more true cases than the second-best model (91, 72, and 135 more than the nontemporal Latest-Value model) at 2, 3, and 4 years. Moreover, the Landmark-Boosting model was clearly better than the other models on calibration that never underestimated the risks (Figure 6), whereas the Stack-Temporal model also seemed to be well calibrated within the first 2 years of DM onset.

### Clinical Implications

Our proposed temporal model will benefit patients with longitudinal data, and the longer we follow up, the better the model can predict the next-year DKD risk by self-adjustment with respect to both the individual’s medical history and population shift over time. The study has three important implications. First, our investigation confirmed that temporal EHR and billing data carry critical information depicting the progression of the patient’s condition, and it is important to choose the appropriate method for incorporating longitudinal data to promote the *predictivity* of modern medicine. Second, by allowing the model to evolve along patients’ landmark times, we not only reduced the biases related to a patient’s exposure within EHR but also simulated a scenario that mirrors the clinical practice for annual screening. Third, rather than prior predictive analyses that were mostly population based [40] or personalized longitudinal models requiring complete patient history [10], our model sought a middle ground, aiming to weave together information at both population and individual levels, for example, the GBM built at each landmark time is an attempt to fit the concurrent population, whereas the carrying over of last individual predictions is for the purpose of preserving personal information.

Our model can continually calculate kidney disease risk for patients with diabetes with automatic collection of new EHR data and improve prediction over time. The ability to precisely stratify patients with diabetes by their renal complication risk in the coming year would merit a variety of potential intervention designs: (1) *nutritional interventions* that differentiate dietary consultation according to relative DKD risk, for example, presenting dietary flyers to all patients with type 2 DM but arranging in-person consultation sessions for those in the high-risk bin with dietitians knowledgeable in CKD diet; (2) *lifestyle interventions* that encourage personalized health-promoting behaviors such as smoking cessation and physical activity at different intensity levels based on their DKD risk; (3) *medication management* by designing targeted strategies according to the risk to encourage patient medication compliance, especially with blood pressure and glucose control medications, and warn patients and physicians against the use of nephrotoxic medications, for example, nonsteroidal anti-inflammatory drugs, unless absolutely necessary for high-risk patients because patients with diabetes are already at a higher risk for developing transient decreases in renal function consistent with acute kidney injury, and nephrotoxic drug exposure can amplify that risk. Moreover, with the DKD risk factor discovery framework developed in our previous work [41], we can further empower the predictive models by outputting explainable risk factors and quantifying their effects on DKD specific to subgroups within different risk bins to better support physicians in designing tailored therapy and management strategies. More importantly, the Landmark-Boosting model almost never underestimated the risk compared with other models, especially among the high-risk group, which is clinically ideal because timely medication management can be effective in protecting high-risk patients from unnecessary harm to the kidney due to the use of nephrotoxic medications.

### Limitations and Future Work

There are several limitations to our work. Disease diagnosis sequence is not necessarily the same as the disease manifestation sequence, which may lead to the underestimation of false-negative rates for DKD in this study. For example, our exclusion criteria may have excluded patients with DKD who visited our hospital for their kidney disease but have not had their diabetes-related information recorded in our EHR yet. In addition, the current design of our model is not robust against population drift because of changes in practice over time or differences in clinical vocabulary and workflow implemented across institutions. To further investigate the generalizability of our model, it is necessary to perform external validations and adequate recalibration based on patients from different sites as well as over calendar years to capture the general population shift and practice change.

Although not the focus of this paper, we further examined the factors that potentially contributed to the superiority of the Landmark-Boosting model. In Multimedia Appendix 1, we present the top 50 important features selected by the Landmark-Boosting model and their varying rankings among the other temporal models. Only a few important variables were common across all models (eg, age at DM onset and creatinine). Most top-ranked factors by the Landmark-Boosting model were less important in the other three temporal models (eg, previous visit to cardiovascular clinic, triglycerides, glucose, and exposure to codeine derivative). Furthermore, we examined the features that may contribute to improving the performance of Landmark-Boosting model over time. As shown in Multimedia Appendix 1, we collected the top 30 important features at year 4 and backtracked their rankings in previous years. For each feature, we calculated the Pearson correlation coefficient between ranking and landmark time to determine if the feature ranking increased/decreased significantly over time. Factors showing improved predictive power over time included cumulative clinical fact counts, previous visit to cardiovascular clinic, systolic blood pressure, triglycerides, and alanine aminotransferase. Built on these preliminary findings, we plan to further characterize and evaluate the changing feature representations over time in our future work.

### Conclusions

This study addressed the problem of underutilization of temporal information in EHR-based predictive models. We proposed a new approach in leveraging the temporal dynamics in EHR to improve DKD prediction and validated it against three state-of-the-art models using the idea of *landmark time* to simulate real clinical utility. Experimental results demonstrated that the proposed Landmark-Boosting model can effectively capture temporal dynamics in EHR without overfitting and further improve on patients with a longer follow-up time.

## Appendix

Multimedia Appendix 1 Variable importance ranking across model and over time.

## Abbreviations

ACR: albumin-to-creatinine ratio
AUPRC: area under precision recall curve
AUROC: area under receiver operating curve
CKD: chronic kidney disease
DKD: diabetic kidney disease
DM: diabetes mellitus
eGFR: estimated glomerular filtration rate
EHR: electronic health record
ESRD: end-stage renal disease
GBM: gradient boosting machine
GFR: glomerular filtration rate
HbA_1c_: glycated hemoglobin
HERON: Healthcare Enterprise Repository for Ontological Narration
PPV: positive predictive value
