# Supplementary material for: Longitudinal Risk Prediction of Chronic Kidney Disease in Diabetic Patients Using a Temporal-Enhanced Gradient Boosting Machine: Retrospective Cohort Study
Source: JMIR Med Inform. 2020 Jan 31;8(1):e15510. doi: 10.2196/15510 (PMC7055762; doi:10.2196/15510)
Supplement: Multimedia Appendix 1 [file medinform_v8i1e15510_app1.docx]

##
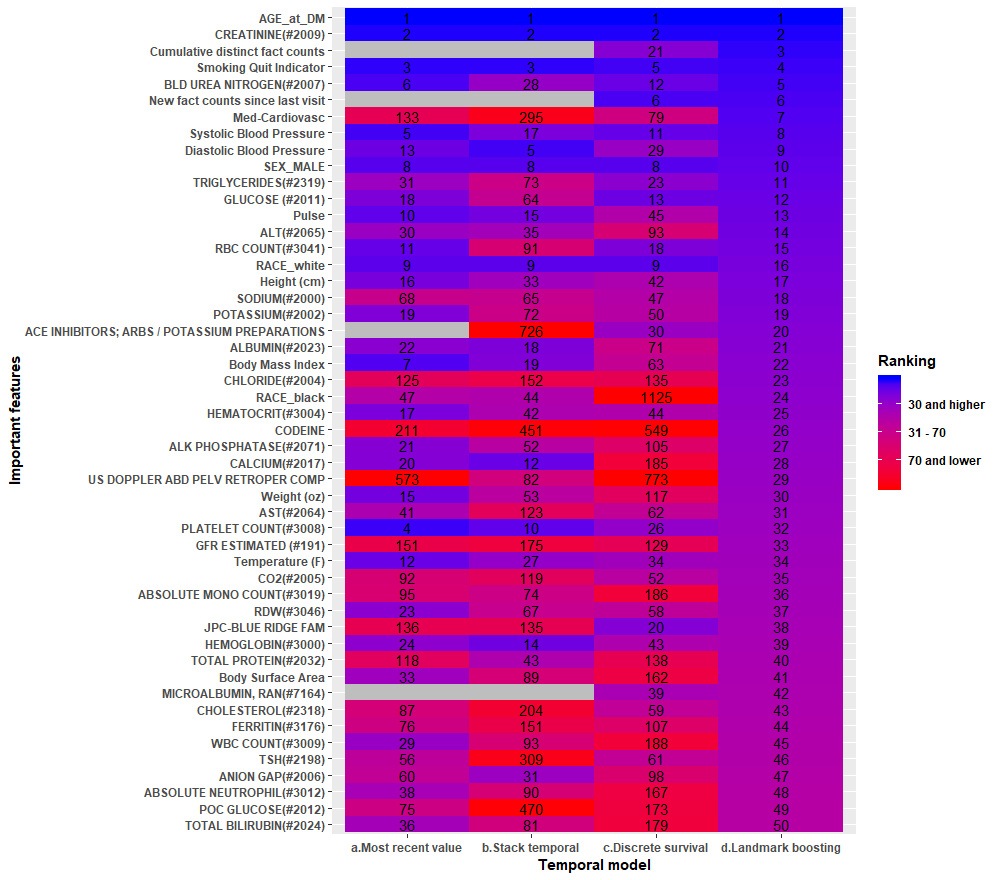
Multimedia Appendix A

\

**Supplemental Figure 1 – heatmap demonstrating the variable importance ranking across different temporal models at year 4 since DM onset.**  Each cell in the heatmap is corresponding to the rank of one feature in one particular temporal model. Features are listed based on their importance rankings in the Landmark Boosting model. The greyed cell suggests that a feature was not selected by the corresponding model.

##
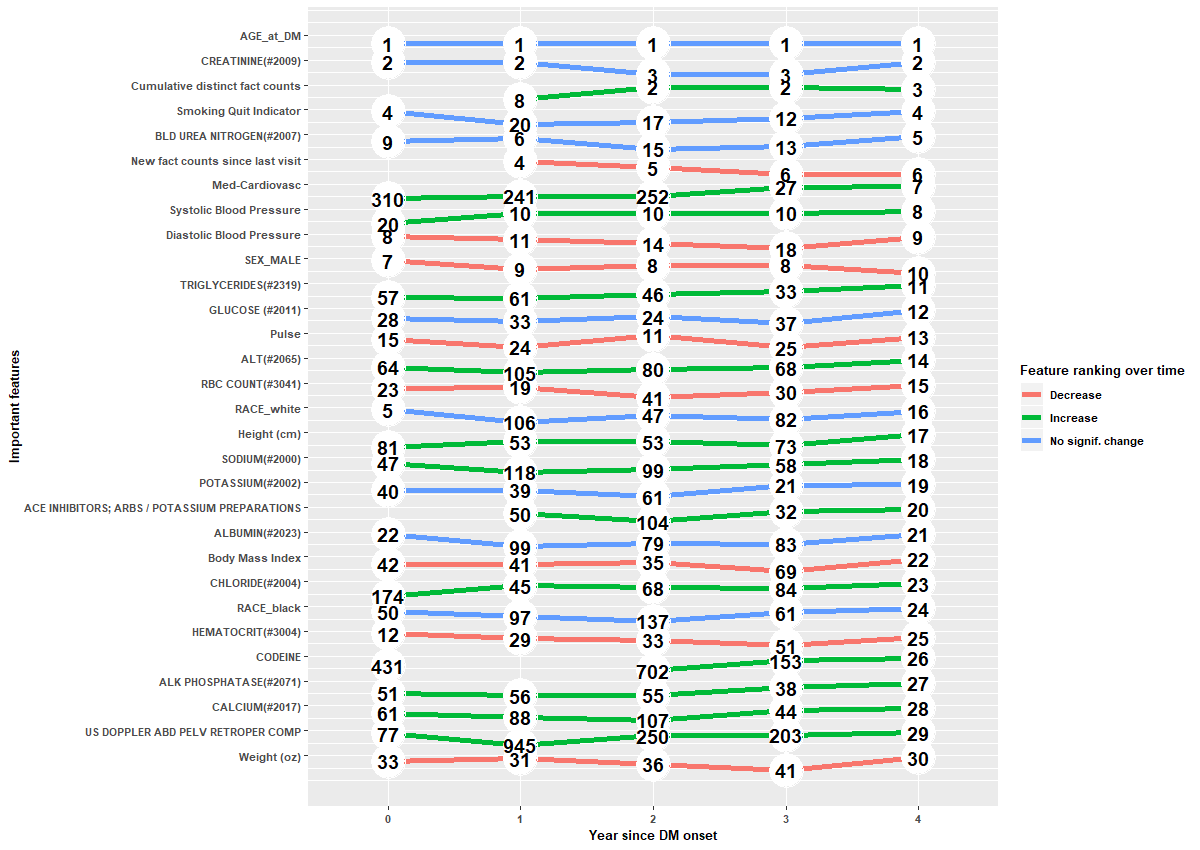
Multimedia Appendix B

**Supplemental Figure 2 – variable importance ranking (top 30) drift over years for Landmark Boosting Model.** Each dot represents the rank of a feature in Landmark Boosting Model at a particular landmark time. For each feature , we calculated Pearson correlation coefficient between ranking and landmark time to identify if there is a significant positive/negative linear association between ranking and time, where: a) a significant positive correlation suggests that the rank decreases over time (“Decrease”); b) a significant negative correlation suggests that the rank increases over time (“Increase”); c) non-significant correlation suggests no significant ranking range over time (“No signif. change”).
